# Building public health capacity in Madhya Pradesh through academic partnership

**DOI:** 10.3402/gha.v7.24839

**Published:** 2014-08-13

**Authors:** Ritika Tiwari, Anjali Sharma, Himanshu Negandhi, Sanjay Zodpey, Nidhi Vyas, Manohar Agnani

**Affiliations:** 1 Public Health Foundation of India, Gurgaon, Haryana, India; 2 State Institute of Health Management & Communication (SIHMC), Gwalior, Madhya Pradesh, India; 3 Food & Civil Supplies and Consumer Protection, Government of Madhya Pradesh, Bhopal, Madhya Pradesh, India

**Keywords:** public health management, capacity building, partnership, Madhya Pradesh, NRHM, academics, health system

## Abstract

Engaging in partnerships is a strategic means of achieving objectives common to each partner. The Post Graduate Diploma in Public Health Management (PGDPHM) partners in consultation with the government and aims to strengthen the public health managerial capacity. This case study examines the PGDPHM program conducted jointly by the Public Health Foundation of India and the Government of Madhya Pradesh (GoMP) at the State Institute of Health Management and Communication, Gwalior, which is the apex training and research institute of the state government for health professionals. This is an example of collaborative partnership between an academic institution and the Department of Public Health and Family Welfare, GoMP. PGDPHM is a 1-year, fully residential course with a strong component of field-based project work, and aims to bridge the gap in public health managerial capacity of the health system through training of health professionals. The program is uniquely designed in the context of the National Rural Health Mission and uses a multidisciplinary approach with a focus on inter-professional education. The curriculum is competency driven and health systems connected and the pedagogy uses a problem-solving approach with multidisciplinary faculty from different programs and practice backgrounds that bring rich field experience to the classroom. This case study presents the successful example of the interface between academia and the health system and of common goals achieved through this partnership for building capacity of health professionals in the state of Madhya Pradesh over the past 3 years.

The current medical education system in India focuses minimally on the development of managerial competencies to address public health needs of the population (1), resulting in medical graduates with poor public health management skills. Ironically, the job profile of such doctors subsequently joining the health system demands knowledge and skills related to public health management. The National Rural Health Mission (NRHM), a flagship program of the Government of India, having recognized this gap, launched a 1-year Post Graduate Diploma in Public Health Management (PGDPHM) in July 2008 to impart public health management knowledge and skills to working health professionals. The conceptualization, design, and implementation of this program was entrusted to the PGDPHM consortium of 11 public health institutes across the country, as a partnership under NRHM. The Public Health Foundation of India (PHFI) has provided leadership to this consortium since its inception. As of 2014, 882 students have enrolled and 723 have graduated from 11 consortium institutes. The State Institute of Health Management and Communication, Gwalior (SIHMC-G) is one of the participating institutions of the consortium. Since 2010, SIHMC-G has been conducting the PGDPHM program in academic partnership with PHFI.

## Program development

The Government of Madhya Pradesh (GoMP) expressed its desire to develop a sustainable, robust, and dynamic health cadre, which is adequately equipped to address the state's public health challenges. To facilitate this ambitious plan, the GoMP and PHFI signed a memorandum of understanding (MoU) whereby PHFI provides technical support to GoMP in conducting the PGDPHM course at SIHMC-G. The SIHMC-G is the apex training and research center for the health department in Madhya Pradesh. It was conceived as part of a national training program under the Sixth India Population Project (IPP_6) in 1990 and became operational from 1993 at Gwalior (2). Its mandate is to improve the efficiency of health personnel for better service delivery and engaging in health system research. As part of the technical support, PHFI assists the state government in overall management and conduction of the PGDPHM program. PHFI ensures that the post-graduate diploma is awarded as per PHFI's guidelines and quality parameters. This partnership has also provided PHFI an opportunity to engage in need-based training and research to address the health concerns related to Madhya Pradesh. To facilitate the program delivery, PHFI has recruited two full-time faculty members at SIHMC-G, with a provision of travel for existing faculty members on a needs basis.

## Program architecture and implementation

PGDPHM is a 12-month residential postgraduate diploma program, which aims at enhancing the capacity of health professionals by imparting 9 months of classroom teaching and 3 months of field-based project work. Enrollment of in-service health care professionals is based on nominations from the state government. Self-sponsored students with a suitable medical or other health-related qualification are also eligible to apply for this program. The GoMP has agreed to nominate at least 50 candidates annually for this program. PGDPHM program architecture and implementation is explained in Box 1.

***Box 1.* PGDPHM program architecture and implementation**

- Post Graduate Diploma program at SIHMC-Gwalior was initiated in 2010.
- This is a Ministry of Health and Family Welfare, Government of India, supported program.
- Program Duration: 12 months from August to July.
- This is a residential community-based program.
- Academic years: 2010–11, 2011–12, 2012–13.
- Program structure: 35 credit points
  - Taught modules (23 credits):
*Management* – Introduction to Public Health Management, Human Resource Management and Organizational Behavior, Financial Management, Management of MCH/RCH, Project Management, Logistic Planning and Drug distribution/Inventory Management.
*Public health* – NRHM, National Health Programs, Health Systems and Health Sector Reforms, Urban Health, Behavioural and Social Science in Health, Quality, Equity and Access to Health Care, Health Communication and Promotion, Health Management Information Systems, Public Health Nutrition, Communicable Diseases, Non-Communicable Diseases, Health Policy and Health Care Planning, Environmental and Occupational Health.
*Analytical skills* – Epidemiology, Demography, Disease Surveillance, Biostatistics, Research Methods and Operations Research, Essential of Health Economics, Health Financing and Insurance.
  - Field-based project work (12 credits): The project work constitutes the applied research component of the program, aimed at exposing the students to ‘real world’ public health problems and their possible solutions by applying their knowledge and analytical skills in the community setting.
- Eligibility:
  - In-service candidates: Bachelor of Medicine, Bachelor of Surgery (MBBS), or Bachelor of Science (BSc) Nursing, with at least 5 years of work experience with Department of Public Health and Family Welfare, Government of Madhya Pradesh.
  - Self-sponsored candidates: MBBS, Bachelor of Dental Surgery (BDS), Master of Social Work (MSW), BSc Nursing, Bachelor of Ayurvedic Medicine and Surgery (BAMS), Bachelor of Homoeopathic Medicine & Surgery (BHMS), Bachelor of Unani Medicine and Surgery (BUMS), Bachelor of Physiotherapy (BPT).

The PGDPHM program comprises a multidisciplinary, health systems–related problem-solving and competency-driven curriculum with a focus on broad areas such as public health, management, and analytical skills. PGDPHM student competencies in the following areas have been designed using a consultative approach: program planning and management; project development and management; monitoring and evaluation; good understanding of national health programs and guidelines; health policy and environment analysis at district, state, and national levels; practical knowledge of conducting primary quantitative and qualitative research including tool development and data collection, data management, and so on; data analysis using statistical packages and report writing, adequate managerial skills including human resource management, financial management, and logistics and drug distribution. The curriculum is based on core and cross-cutting competencies that are expected to be acquired by a public health manager.

In addition to two full-time faculty members posted at SIHMC-G, another faculty of PHFI serves as a visiting faculty on an as-needed basis to complement the gap in the existing human resource for the PGDPHM program. Faculty members posted at SIHMC-G have a multidisciplinary professional background; are internationally trained with strong training, practice, and research skills; and have several peer-reviewed publications to their credit.

## Features of the PGDPHM program

One of the strengths of the PGDPHM program is that its content and pedagogy are centered around the objectives of the NRHM and this program involves all key stakeholders through all stages of the program cycle, that is, from program design to its evaluation (3). For example, the design of the program architecture, curriculum contents, and evaluation were jointly agreed upon with discussions between the Ministry of Health, NRHM, public health experts, academicians, and the partner institutions. The curriculum design was rigorously peer-reviewed by public health experts from health systems as well as academics. The academic team also regularly contacts the employers of these graduates and seeks their feedback on the performance of these graduates. Student feedback is also regularly sought on their teaching – learning experiences as a partners’ program.

The multi-disciplinary nature of the PGDPHM program is reflected in wide-ranging student profiles, diverse course curriculum, and varied educational backgrounds/experience of the faculty. The competency-driven curriculum of the PGDPHM program ensures enhancement of analytical and program management skills of the students. The PGDPHM curriculum is reviewed and updated every year through a consultative process of the partner institutes. Another important factor contributing toward the uniqueness of this program is the use of innovative pedagogy from the Indian public health teaching perspective. Unlike traditional approaches toward public health education in medical schools across the country (4), the PGDPHM program applies the principles of adult learning for improving the teaching–learning experience. Suggested pedagogy includes a predominant use of the following approaches: article review, collaborative learning, seminar, panel discussion, participant assignments, visits to organizations, group discussion, case study, lecture, field work, field projects, report writing, journal club, and so on. The direct linkage to health systems’ capacity building is another strength of this program which distinguishes PGDPHM from other health management courses being offered in the country. The quality of this program is managed and monitored through robust quality assurance systems and standard operating procedures so as to provide high-quality education and fully satisfy the needs and expectations of the students and other stakeholders.

## Objectives

This case study aims to examine the PGDPHM program conducted jointly by the PHFI and the GoMP at SIHMC-G which is an apex training and research institute of the state government for health professionals.

## Results

The activities being carried out through this partnership have resulted in achieving the objective of growth and capacity enhancement of existing public health institutions in the country. Since 2010, 114 in-service candidates (working in the health sector in central and state governments) and self-sponsored participants (graduates/post-graduates in medicine, AYUSH, dental, nursing, pharmacy, health sciences, etc.) have graduated from this institution. The majority of them (57%) were Bachelor of Medicine, Bachelor of Surgery (MBBS) graduates followed by 21% nursing graduates. Of these students, 79% were in-service, government-sponsored candidates and 21% were self-sponsored candidates.

In the year 2011, the GoMP re-positioned almost all government-sponsored candidates (doctors and nurses) to appropriate positions with managerial job responsibilities at the district and state level. Of these, several medical officers who were earlier posted at various Primary Health Centres (PHCs) and Community Health Centres (CHCs) in Madhya Pradesh were allocated to the position of district program managers under NRHM. Similarly several staff nurses working at district level CHCs/hospitals were posted as in-charge program officers at the state level. One important indicator of success of the initiative is that the majority of PGDPHM graduates (85%) are working within the government health system irrespective of their sponsorship status. A similar exercise was undertaken for assigning appropriate positions with managerial job responsibilities for the students graduating in the academic years 2011–2012 and 2012–2013. The performance of these graduates after their placement within the health department was also assessed as a part of the program evaluation undertaken in 2012 (5). One of the recommendations of this report was that the state health systems should design an enabling environment and provide incentives to ensure proper utilization of skills of the PGDPHM graduates (5).

## Discussion

Partnerships in public health offer an opportunity to share resources across organizations and health system. They also have the potential for promotion of interprofessional education^1^ (6) and transprofessional education^2^ (7) by breaking down professional silos and enhancing collaborative and non-hierarchical relationships in effective teams (8). Institutions can collaborate together by leveraging each other's strengths and undertake joint programs. The Lancet Commission Report on Health Professions for the 21st Century states that the cross-institutional collaborations can link educational centres to policies and practices, while offering partner governments, non-governmental organizations, businesses, and media organizations complementary academic resources. Students can be offered training, internships, or work–study experiences in such collaborating institutions, and the partner group can capitalize on the faculty resources of the educational institution (8).

The PGDPHM program was designed to bridge the gap in public health managerial capacity among health professionals in the context of the NRHM. This program is a unique model of partnership between academic institutions and health system. This flagship initiative also strove to strengthen the capacities of partner institutions and networks of professionals to take the lead in designing, adapting, and sustaining innovative capacity-building measures. For the PGDPHM program being offered at SIHMC-G, focussed approach of goal-oriented management team and staff proved to be an important conduit to success of the program. The PHFI-GoMP partnership has brought strong academia and state health systems to interface with each other, where classroom teaching can be translated to practical applications in the field. The health systems connectivity of the program has helped students to relate classroom learning to real-life situations. The program has a multi-discipliner curriculum (Box 1) which is taught by academicians/experts with professional qualifications and experience in these areas. This contrasts with the pre-dominant faculty qualifications across medical schools which are responsible for training the largest number of public health professionals across India. The syllabus is competency-based and meets the 21st century reforms, which talks of systems strengthening through a competency driven curriculum (3). This gave ample opportunities to the students to engage in critical thinking and deliberating in new ways to bridge the gap in existing flaws in the health care system and thereby improve health outcomes.

Another strength of the program is that the program batches comprise students from several disciplines, thus inter-professional learning was conducive in enhancing the importance of team-based and problem-based learning. GoMP has been nominating the medical and nursing professionals to the PGDPHM program which ensures an inter-professional learning environment for the scholars. Through this initiative, the state government is also assuring suitable placement opportunities for these trained health professionals in the health systems post-completion of the PGDPHM program. The Institute of Medicine (IOM), the World Health Organization (WHO), and other key organizations have recognized that a solution to today's complex health problems urgently requires collaboration among health professionals from multiple disciplines, which is very much reflected in the program philosophy (9, 10).

In 2012, on the Government of India's request, an evaluation of PGDPHM was conducted by PHFI with support from Wellcome Trust Capacity Building Strategic Award (5). This evaluation indicated that PGDPHM has contributed significantly toward strengthening health systems in India (5).

This important partnership has evolved successfully in the past 3 years and some systematic efforts have been directed to make the state health system stronger. Moreover, we have learned to adapt the program contents, particularly its implementation by linking the teachings to the context of the health system and public health challenges confronting Madhya Pradesh. Madhya Pradesh, being a high focus state, is receiving increased attention from the ministry as well as the state health department. There is an opportunity for the participants to bring in their experiences to the classroom and for teachers to contextualize learnings to the Madhya Pradesh context. Earnest efforts are being undertaken to revamp the physical and IT infrastructure to ensure educational resources are maximized. Also, state faculty's potential toward contribution for the long-term academic programs has been enhanced.

A strong public health infrastructure that hopes to address the wide range of needs within a community requires sound relationships and active collaboration between academia and the current as well as future public health workforce. An effective engagement with the government sector can be instrumental in attending to the issues of equity and quality improvement of the services provided along with dealing with the issues of access and responsiveness of the system. Effective academic–community collaborations have the potential to facilitate a broad-based appreciation of public health among students via a wide array of public health curricula and applied experiential learning opportunities in public health settings (11). Collaboration, a potentially powerful instrument of academic systems, describes the opportunities to enhance educational quality and productivity through sharing of information, academic exchange, pursuit of joint work, and synergies between institutions (12). Collaboration can serve many purposes, deploy several instruments, and takes place at different levels. It ultimately involves the relationship between individuals, but it can be structured and sustained through formalized institutional arrangements that promote, finance, and sustain relationships over time. The institutional purposes in education, research, and service can be advanced through sharing of curricula, exchange of faculty and students, collaborative research, and other activities. Building the capacity of health care professionals is paramount for health systems to strengthen and fortify such partnerships and holds great promise to transform health systems.

## Footnotes

1 Interprofessional education occurs when students from two or more professions learn about, from, and with each other to enable effective collaboration and improve health outcomes.

2 Transprofessional learning is described as interprofessional learning (exploring the subject from one's own and other professional angles), based on the experience of the real world.
